# Sub-Clinical Cognitive Decline and Resting Cerebral Blood Flow in Middle Aged Men

**DOI:** 10.1371/journal.pone.0169912

**Published:** 2017-01-17

**Authors:** Otto Mølby Henriksen, Naja Liv Hansen, Merete Osler, Erik Lykke Mortensen, Dorte Merete Hallam, Esben Thade Pedersen, Michael Chappell, Martin Johannes Lauritzen, Egill Rostrup

**Affiliations:** 1 Functional Imaging Unit, Dept. of Clinical Physiology, Nuclear Medicine and PET, Copenhagen University Hospital Rigshospitalet Glostrup, Glostrup, Denmark; 2 Center for Healthy Aging, University of Copenhagen, Copenhagen, Denmark; 3 Dept. of Clinical Physiology, Nuclear Medicine and PET, Copenhagen University Hospital Rigshospitalet Blegdamsvej, Copenhagen, Denmark; 4 Dept. of Clin. Physiology and Nuclear Medicine, Copenhagen University Hospital Herlev, Herlev, Denmark; 5 Dept. of Public Health, University of Copenhagen, Copenhagen, Denmark; 6 Research Center for Prevention and Health, Copenhagen University Hospital Rigshospitalet Glostrup, Glostrup, Denmark; 7 Dept. of Radiology, Copenhagen University Hospital Rigshospitalet Glostrup, Glostrup, Denmark; 8 Danish Research Centre for Magnetic Resonance, Copenhagen University Hospital Hvidovre, Hvidovre, Denmark; 9 Institute of Biomedical Engineering, University of Oxford, Oxford, United Kingdom; 10 Oxford Centre for Functional MRI of the Brain, Nuffield Dept. of Clinical Neurosciences, University of Oxford, Oxford, United Kingdom; 11 Dept. of Neuroscience & Pharmacology, University of Copenhagen, Copenhagen Denmark; 12 Dept. of Clinical Neurophysiology, Copenhagen University Hospital Rigshospitalet Glostrup, Glostrup, Denmark; "INSERM", FRANCE

## Abstract

**Background:**

Although dementia is associated with both global and regional cerebral blood flow (CBF) changes, little is known about cerebral perfusion in the early pre-clinical stages of cognitive decline preceding overt cognitive dysfunction. The aim of this study was to investigate the association of early sub-clinical cognitive decline with CBF.

**Materials and Methods:**

The study participants were recruited from a cohort of Danish men born in 1953. Based on a regression model we selected men who performed better (Group A, n = 94) and poorer (Group B, n = 95) on cognitive testing at age 57 than expected from testing at age 20. Participants underwent supplementary cognitive testing, blood sampling and MRI including measurements of regional and global CBF.

**Results:**

Regional CBF was lower in group B than in group A in the posterior cingulate gyrus and the precuneus. The associations were attenuated when corrected for global atrophy, but remained significant in regions of interest based analysis adjusting for regional gray matter volume and vascular risk factors. No influence of group on global CBF was observed.

**Conclusions:**

We conclude that early sub-clinical cognitive decline is associated with reduced perfusion in the precuneus and posterior cingulate gyrus independently of regional atrophy and vascular risk factors, but cannot be statistically separated from an association with global atrophy.

## Introduction

Dementia is a leading cause of age-related disability worldwide, and the number of demented people is expected to triple by the year 2050 [1]. Although overt dementia or mild cognitive impairment may be preceded by a phase of sub-clinical cognitive decline, the significance and the mechanisms of early cognitive decline in clinically normal individuals have not been established.

Because effective treatment is lacking, interest has gathered around preventive or disease modifying measures at the pre-clinical stage, and thus, the identification of modifiable risk factors and biomarkers of early disease have become areas of great interest [2]. Mounting evidence has suggested that vascular pathology and dysfunction are involved in the development of Alzheimer’s disease, narrowing the gap between primarily vascular and neurodegenerative brain diseases [3].

Imaging of regional cerebral blood flow (CBF) patterns provides direct information on brain tissue perfusion, but is also used as a marker of brain tissue function and integrity and has been widely used clinically in the evaluation of patients with cognitive dysfunction [4]. Regional CBF changes may be present even in asymptomatic individuals at risk of dementia [5–7], indicating the ability of regional CBF measurements also to detect subclinical pathology. Further, reduced global CBF has been demonstrated not only in patients with clinical dementia, but has also been associated with structural signs of brain aging and cognitive decline in non-demented individuals [8–11].

Together, the above observations suggest that cerebral hypoperfusion may be present in the early stages of cognitive dysfunction. However, altered CBF may also reflect the possible effect of vascular risk factors [12;13] and brain atrophy [14;15], and should therefore be accounted for in the analysis. The aim of the study was to test the hypothesis that early cognitive decline is associated with decreased regional or global CBF. We furthermore, investigated whether the CBF decline was associated with known vascular risk factors.

Most studies correlating brain function imaging with cognitive function have not taken early life cognitive function into account and have investigated the relationship with cognitive function at the time of the study rather than the effect of cognitive decline per se. In the present study, we report on the findings of a magnetic resonance imaging (MRI) study of middle-aged men with and without late midlife sub-clinical cognitive decline. The study applies modern MRI techniques for absolute quantification of regional CBF [16;17], and by measuring also global CBF using phase contrast mapping [10] and hemoglobin, the validity of such measurements could be verified.

## Materials and Methods

### Participants

Participants were recruited from The Metropolit Danish Male Birth Cohort [18;19], which includes all boys born in 1953 in the Copenhagen metropolitan area. The procedure for selecting participants has been described in details previously along with functional MRI data from the same selection of cohort members [20]. In brief, cognitive testing was performed at approximately 20 years of age as a part of the military draft board assessment program using the Børge Priens Prøve (BPP) test [21]. In 2009–2010 at age approximately 56 years, a total of 1985 members of the cohort were cognitively re-assessed using the Intelligenz-Struktur-Test 2000 R (IST) as part of the Copenhagen Aging and Midlife Biobank project (CAMB) [19]. The CAMB version of the IST comprises three subtests, verbal analogies, number series, and sentence completion [22], very similar to subtests included in the BPP.

To identify those who performed relatively poorer on re-assessment than predicted from the early life cognitive score, a regression analysis was carried out with BPP score as the explanatory variable and IST score as the dependent variable. The two test scores were highly correlated (r = 0.71, p<0.0001). Each participant’s standardized residual was considered a measure of the relative cognitive change from early adulthood. To minimize the effects of individuals with potentially unreliable or extreme test scores, those with absolute standardized residuals >3 were excluded. In order to take into account the normal variability of cognitive test scores and to maximize the ability to detect any influence of cognitive decline, we identified two groups: Group A consisting of cohort members with the largest positive residuals serving as a control group, and Group B with the largest negative residuals considered as those with apparent sub-clinical cognitive decline. Cohort members were invited to participate in the present study until at least 100 participants were included in each group.

**Fig 1:**
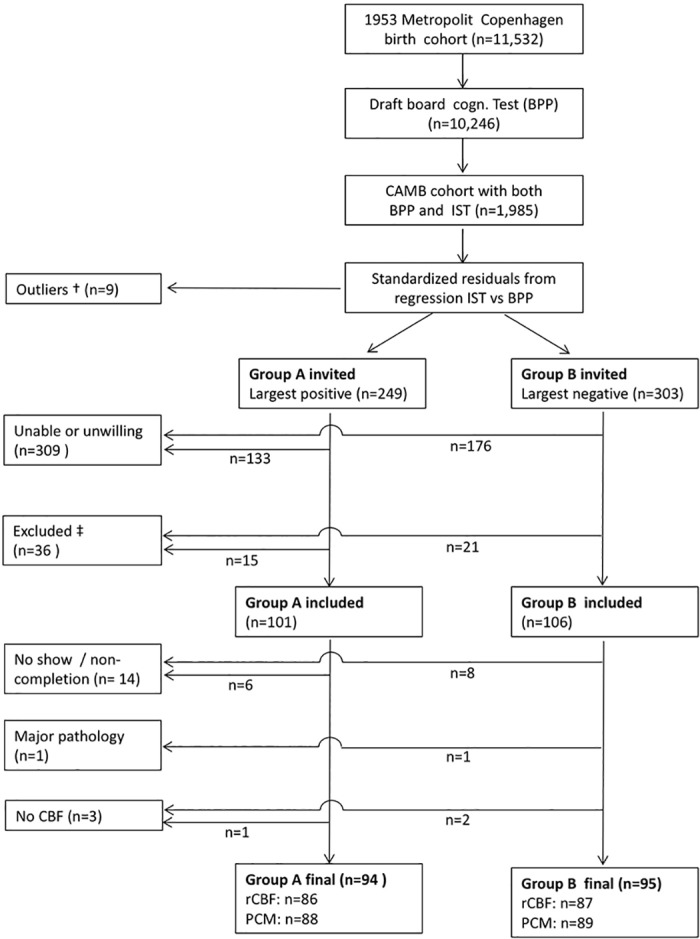
Selection of participants. Flowchart showing selection of the current study sample from the original 1953 Metropolit Copenhagen Birth Chohort. † defined as standardized residual exceeding ±3 ‡ Excluded according to predefined exclusion criteria (alcohol and drug abuse, major psychiatric and neurologic disease, major structural brain lesions, and contraindications to MRI).

Data collection took place in 2010–2012 (at subject age 58±0.7 years). A flowchart showing the selection of participants is shown in Fig 1. In short 48% of those invited accepted to participate, and of these 36 persons were excluded according to predefined exclusion criteria (alcohol and drug abuse, major psychiatric and neurologic disease, major structural brain lesions, and contraindications to MRI), while 18 persons were missing MRI data for other reasons, leaving 189 data sets with either regional or global perfusion. One regional CBF map was discarded due to poor quality. In the final study population (94 in group A and 95 in group B) regional CBF maps were available in 173 (86 in group A and 87 in group B) and total flow measurements in 177 participants (88 in Group A and 89 in Group B). Both measurements were available in 162 participants (81 in Group A and 81 in Group B).

The study was approved by the regional ethics committee (Scientific Ethics Committee of The Capital Region, Protocol no. H-3-2010-016), following the standards of The National Committee on Health Research Ethics. The experiments were conducted in accordance with the Helsinki Declaration and all participants gave written informed consent.

### Cognitive testing

To confirm the group difference at follow-up, and to characterize the cognitive performance of the participants in more details, a comprehensive neuropsychological test battery was administered as close as possible to the MRI—often on the same day and for 90% of the participants within the same week. The tests (presented in Table 1) focused on visual and verbal learning and memory, attention, processing speed, as well as overall global function. The test battery included classical neuropsychological tests, as well as tests from the Cambridge Neuropsychological Test Automated Battery (CANTAB), and was administered by staff who were trained and supervised by an experienced neuropsychologist, and were blinded to the BPP and IST scores. Measurements of blood pressure, weight and height, as well as other demographic data were also obtained, generally on the same day as the cognitive examination.

**Table 1:** Study population characteristics.

	No decline		Decline	
	(Group A, n = 95)		(Group B, n = 94)	
	Median	Range	Median	Range
*Risk factors*				
P-homocysteine, μmol/l	8.4	(4.4–25.5)	8.6	(5–17.4)
P-cobalamin, pmol/l	352	(176–738)	339	(173–639)
Total cholesterol, mmol/l	5.5	(3.5–7.8)	5.5	(3.2–8.4)
LDL:HDL	2.59	(1.00–5.6)	2.42	(0.42–5.56)
BMI, kg/m^2^	26	(20–43)	27	(20–35)
MAP, mmHg	105.7	(80.3–126.3)	105.3	(74.3–156. 7)
Current smoker, n	18		17	
Package-years	4.3	(0–78)	2.5	(0–126)
Number of APOE4 alleles, n = 0/1/2	70/19/0		61/28/3§	
Education length, years	16	(8–23)	13**	(7–22)
*Cognitive testing*				
BPP test, score	48	(16–61)	46	(28–68)
IST, score	44	(19–55)	21**	(9–35)
MMSE, score	30	(26–30)	29*	(25–30)
ACE, score	97	(85–100)	93**	(70–100)
Trailmaking A, sec.	31	(18–80)	32	(19–62)
Trailmaking B, sec.	67	(35–140)	78**	(42–465)
15 word paired ass. learning, no. errors	6	(0–29)	14**	(1–33)
15 word paired ass. recall, no. errors	3	(0–12)	6**	(0–15)
Visual, paired ass. learning, no. errors	18	(9–26)	16**	(9–24)
Rapid visual processing, score†	0.941	(0.789–1)	0.898**	(0.742-.990)
5 choice movement time, msec†	371.3	(245.3–607)	369.4	(240.5–742.8)

† from the CANTAB test battery.

Significance test for group differences

*p<0.01 and

**p<0.001 using Mann-Whitney test or t-test where appropriate

§ p = 0.057 using Fisher’s exact test (and p = 0.069 when analyzing APO4 positive vs negative).

Abbreviations: LDL = low density lipoprotein, HDL = high density lipoprotein, BMI = body mass index, MAP = mean arterial blood pressure, APOE = apolipoprotein E, BPP = Børge Priens Prøve, IST = Intelligenz-Struktur-Test, MMSE = mini-mental state examination, ACE = Addenbrooke’s cognitive examination, CANTAB = Cambridge neuropsychological test automated battery.

### Laboratory testing

Non-fasting blood samples were drawn from a cubital vein and analyzed for hemoglobin, homocysteine, cobalamine, plasma lipids and APOE genotype. LDL:HDL ratio was calculated as a measure of dyslipidemia. APOE genotype (rs429358 and rs7412 variants) was determined by pyrosequencing using the PyroMark Q24 system (Qiagen, Hilden, Germany).

### MRI study

#### MRI experiments

All MRI measurements were performed on a 3.0 T Philips Intera Achieva (Philips Medical Systems, Best, the Netherlands) using a 32 element phased array receive head coil and multitransmit parallel RF transmission. In all participants the following MRI sequences were acquired during resting conditions: 1) a high resolution 3D T1 weighted gradient echo sequence (TR/TE = 6.9/700 ms, flip angle = 9°, voxel size 1.1 x 1.1 x 1.1 mm) 2) a T2 weighted (TR/TE = 1300/12 ms, flip angle = 90°, 32 slices, voxel size 1.8 × 1.8 × 9.5 mm), and 3) a T2 FLAIR (TR/TE = 11000/125 ms, flip angle = 90°, 6 slices, voxel size 0.45 × 0.45 × 4.5 mm).

Phase contrast mapping (PCM) was used to measure volume flow in basilar and the internal carotid arteries (ICAs), and normalizing total flow to brain size, mean global CBF can be calculated [10]. ECG gated (retrospective gating, 20 frames/cycle) PCM measurements were obtained with a matrix of 320x320 (TR/TE = 12/7 ms, flip angle 10°, voxel size 0.75x0.75x8mm) with a V_enc_ of 100 cm/s.

**Fig 2:**
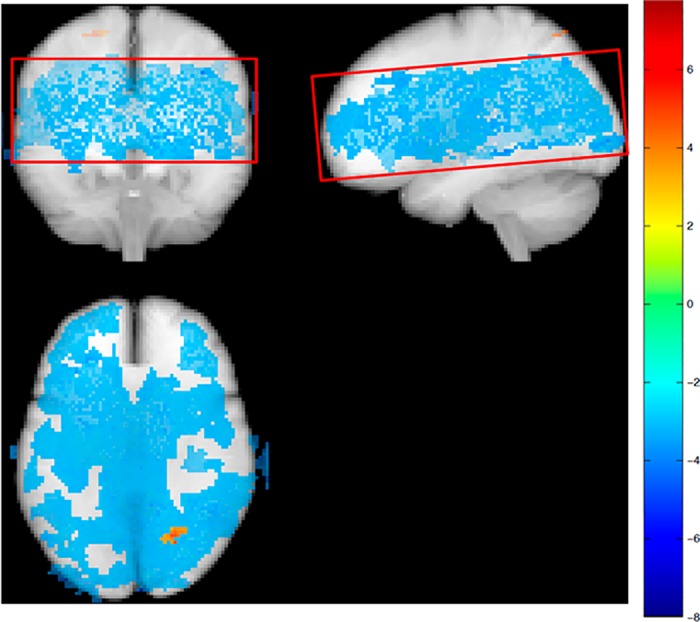
Effect of hemoglobin on regional CBF. Glass brain representation of voxels in absolute CBF maps with significant (p<0.001) negative correlation with blood hemoglobin. Brain volume covered by the ASL measurements is shown in the red box.

Regional CBF maps were obtained using a multi-slice, multiple inversion time (TI) pulsed arterial spin labelling (ASL) sequence (matrix 80x80, voxel size 3x3x6 mm, gap 1.5 mm, TR/TE/ΔTI/TI1 = 4000/22/300/40 ms, flip angle 35°/11.7°, SENSE 2.5, 84 averages [48@Venc = 4cm/s, 24@Venc = ∞, 12 low flip angle]) [23]. Seven transaxial slices were acquired parallel to the lower edges of the corpus callosum. The labelling slab was placed to cover the circle of Willis in order to minimize the possible influence of high flow velocities on bolus length [24]. Accordingly, the most inferior parts of the brain were not covered. (Fig 2)

#### Post-processing

All MRI data were converted into NIfTI format and analyzed using in-house software for Matlab version 7.9 (The MathWorks Inc., Natick, MA) except when indicated otherwise.

Processing of PCM measurements was performed as previously described [25]. The procedure involves an automatic pixel-wise phase correction procedure and subsequent manual assignment of regions of interest for both ICAs and the basilar artery. Volume flow in each vessel is calculated by multiplying mean velocity with vessel area and integrating over time. Mean global CBF is calculated as total brain flow divided by brain volume and reported in ml/100g/min assuming a tissue density of 1 g/ml.

Regional CBF maps were calculated using the FSL (FMRIB Software Library, www.fmrib.ox.ac.uk/fsl) QUASIL tool applying model-based quantitation, and adjusting the T1 value of arterial blood in each subject according to the individual hemoglobin value [16]. This procedure produces maps of absolute perfusion in ml/100g/min as well as the estimated tissue relaxation rate R1. The perfusion maps were spatially normalized to the MNI standard brain provided by FSL using an intermediate step in which the R1-images were co-registered to the individual high-resolution anatomical scan. Both steps were carried out using standard linear co-registration with 12 degrees of freedom. Additionally, relative CBF maps were produced by normalizing absolute CBF maps to a mean brain perfusion value of 50 ml/100g/min.

The FSL BET and FAST tools were used to segment the 3D T1 weighted scan and the resulting cerebrospinal fluid (CSF), gray matter and white matter probability maps were used to calculate the total brain tissue volume (V_tot_), CSF volume (V_csf_) and brain parenchymal fraction (BPF) calculated as

$$BPF=V_{tot}/(V_{tot}+V_{csf})$$

In addition, ventricular volume was determined using FSL Sienax. Structural scans were reviewed by an experienced neuroradiologist for pathology and severity of white matter lesions using a modified Fazekas’ rating scale [26].

### Statistical analysis

Group differences were analyzed using Mann-Whitney test for non-normally distributed variables, t-test for normally distributed variables (before or after logarithmic transformation), or Fisher’s exact test for categorical variables.

#### Voxel-based analysis

To identify group differences in regional CBF, voxel wise analysis of regional CBF maps was initially performed by applying a general linear model to the spatially normalized CBF maps. The analysis was performed using in-house software written in Matlab in order to take into account the slightly non-overlapping brain coverage between participants. Thus, data usage was optimized by including voxels in standard space, even if not all participants contributed data to that point. Both absolute and relative CBF maps were analyzed. Edge-effects were avoided by excluding voxels with less than 10 subjects contributing; all central regions represented 150 subjects or more.

To identify relevant covariates a three-step approach was applied. First, CBF maps were analyzed in a model including only group and hemoglobin to identify areas of interest for subsequent ROI based analysis. Secondly, relevant candidate covariates were investigated in ROI based analysis (see below). Finally, relevant covariates identified in the ROI based analysis were included in the final models. To assess the influence of global atrophy, models both with and without BPF were analyzed. Data were checked for outliers using voxel wise calculation of Cook's distance.

In order to control for multiple comparisons, and to take into account spatial correlation within the brain, we calculated the Threshold Free Cluster Enhanced (TFCE) index [27], and statistical testing was performed using permutation based inference [28] and 5000 permutations. We considered findings significant at a corrected p-level of 0.05.

#### Region of interest based and whole brain analysis

In areas identified in the initial voxel-based analysis as showing group difference, average CBF values and gray matter volumes were extracted from corresponding anatomical regions of interest (ROIs) as defined in the Automatic Anatomical Labelling (AAL) standard system [29].

Cognitive group was considered the main explanatory variable of interest, and included in multiple regression models along with the other predefined covariates of interest (hemoglobin and ROI gray matter volume) and other potential vascular risk factor predictors (number of APOE4 alleles, BMI, mean arterial blood pressure, LDL:HDL ratio and smoking pack years) with ROI CBF as the dependent variable. A backwards stepwise model selection was applied to identify the influence of all potential vascular risk factor predictors. According to this procedure, homocysteine was the only vascular risk factor meeting a p-value criterion of 0.05, and was included in the final models. A similar approach was applied to global CBF values replacing gray matter volume with BPF in the analysis.

Mean gray matter CBF was calculated by averaging all cortical regions. For validation purposes, the correlations of absolute mean gray matter CBF with global CBF measured by PCM, and of both with hemoglobin were analyzed by simple linear regression and calculating Pearson’s correlation coefficient.

Except for voxel based analysis, all statistical analysis was performed using STATA 13 SE (StataCorp, College Station, CA).

## Results

### Study population

Study population characteristics are presented in Tables 1 and 2. No differences between the two groups were observed with regards to any of the vascular risk factors. On cognitive testing at age 20, the two groups did not differ, but Group B performed significantly poorer on most cognitive tests at follow-up. While Group A comprised the upper 15% and Group B the lower 15% of the distribution of residuals, there was a considerable overlap in the IST score with Group A covering the upper 87% and Group B covering the lower 64% of the distribution.

**Table 2:** Whole brain magnetic resonance imaging results.

	No decline	Decline
	(Group A, n = 95)	(Group B, n = 94)
	Median (range)	Median (range)
Total flow (ml/min)†	665 (247–1204)	620 (285–1264)
Brain volume (ml)	1225 (1040–1468)	1224 (1052–1438)
Mean global CBF (ml/100g/min)†	54.2 (20.4–98.2)	53.2 (25.3–101.3)
Gray matter CBF (ml/100g/min) ‡	58.7 (34.3–97.0)	55.9 (30.0–92.8)
Ventricular volume (ml)	35.5 (19.4–66.4)	40.1 (22.7–92.3)*
BPF (%)	76.1 (72.9–80.9)	75.9 (69.1–80.1)
Fazekas’ score (0/1/2/3)§	61/24/6/0	64/22/5/1

Significance test for group differences

* p<0.05 using Mann-Whitney test

† data from 177 participants

‡ data from 173 participants

§ data from 186 participants.

Abbreviations: CBF = cerebral blood flow, BPF = brain parenchymal fraction

Ventricular volume was higher in Group B compared to Group A (Table 2). Otherwise the two groups did not differ with regard to whole brain MRI measures.

### Voxel based analysis

According to outlier analysis, one influential finding was detected, but the overall pattern of results and significance was essentially unchanged whether leaving this data set out or not. We therefore present the results including all data.

**Fig 3:**
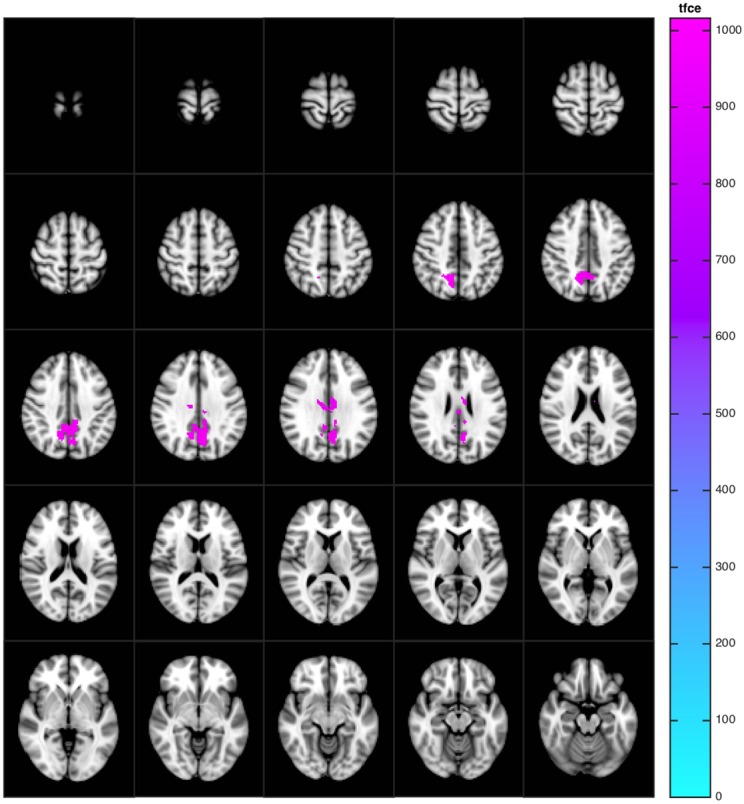
The relationship of group with regional cerebral blood flow. Voxels in absolute cerebral blood flow maps with significant (p<0.05, corrected for multiple comparisons) negative correlation with group (Group B<Group A), adjusted for homocysteine and hemoglobin.

**Fig 4:**
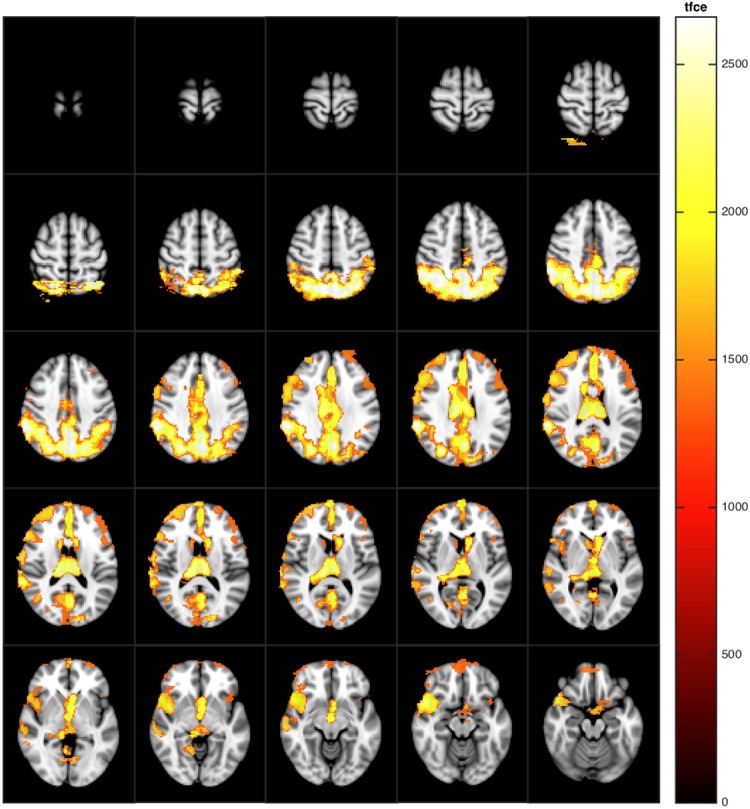
The relationship of brain parenchymal fraction with regional cerebral blood flow. Voxels in cerebral blood flow maps with significant (p<0.05, corrected for multiple comparisons) positive correlation with brain parenchymal fraction, adjusted for group, homocysteine and hemoglobin.

Voxel-based analysis of CBF maps showed that perfusion in the precuneus and in the posterior cingulate gyrus was lower in Group B compared to Group A, also after adjusting for multiple comparisons. (Fig 3) The same group difference was also found using relative CBF maps (not shown) although the number voxels significantly associated with group was lower in relative CBF maps compared to the absolute CBF maps (577 vs 1234 voxels). Adjusting also for the influences of BPF (Fig 4), no significant group differences could be demonstrated. No areas of increased perfusion were identified in group B compared to group A.

### Regions of interest based analysis

The results of the multiple regression analysis are presented in Table 3. Regional as well as global CBF were analyzed in linear models with group, homocysteine and hemoglobin as independent variables. For the ROI based analyses the gray matter density in the same ROI was used as a regressor, while for the global CBF the BPF was used. ROI CBF was lower in Group B compared to Group A, both in the posterior cingulate gyrus and in the precuneus. The analysis also showed that absolute CBF decreased with increasing homocysteine in both regions, although significant only in the precuneus. Including all vascular risk factors in the model, the group effect remained significant (p<0.05 in both regions), but no influence of homocysteine was observed. Also, no significant interaction between group and homocysteine upon CBF was observed in ROI based analysis. Similar effects were observed when analyzing relative CBF maps, although the effects tended to be smaller and statistically less significant. Including educational length or APOE4 status did not change the observed association of group with ROI CBF, and neither of these two covariates were associated with ROI CBF.

**Table 3:** The association (β coeffiecents) of subclinical cognitive decline with regional and global cerebral blood flow.

	Absolute CBF				Normalized CBF				Global CBF	
	Precuneus		Posterior cingulate		Precuneus		Posterior cingulate			
Cognitive decline (Group B-A)	-5.73**	[-9.96,-1.51]	-7.02*	[-12.36,-1.68]	-3.13*	[-5.75,-0.51]	-3.68‡	[-7.41,0.05]	-1.51	[-5.37,2.34]
Hemoglobin (per mmol/l)	-8.38***	[-12.02,-4.74]	-9.33***	[-13.86,-4.80]	-0.56	[-2.81,1.70]	-0.67	[-3.84,2.49]	-8.21***	[-11.49,-4.93]
Homocysteine (per μmol/l)	-0.883*	[-1.70,-0.066]	-0.913†	[-1.950,0.124]	-0.300	[-0.806,0.207]	-0.262	[-0.987,0.462]	0.057	[-0.693,0.806]
ROI GM volume (per ml)	26.6	[-41.7,94.9]	5.63	[-50.4,61.7]	70.16**	[27.8,112.5]	32.3	[-6.86,71.42]	-	
BPF	-		-		-		-		133.0*	[12.5,253.6]

In all models CBF is treated as dependent variable, and group (cognitive decline) as main independent variable, while homocysteine, hemoglobin and gray matter volume (or BPF for global CBF) are covariables. Abbreviations: CBF = cerebral blood flow, ROI GM = region of interest gray matter, BPF = brain parenchymal fraction.

* p <0.05

** p<0.01

*** p<0.001

†p = 0.084

‡ p = 0.053.

### Global CBF

No effects of group or homocysteine on global CBF could be demonstrated, but the multiple regression model (Table 3) did show a significant positive association of global CBF with BPF.

Absolute mean gray matter CBF from ASL measurements was significantly correlated with mean global CBF from PCM measurements (0.38 [95% CI: 0.24 to 0.52], r = 0.39, p<0.001). Both mean gray matter CBF and global CBF decreased with increasing hemoglobin (-7.2 [95% CI: -10.3 to -4.0] ml/100g/min per mmol/l, r = -0.11, p<0.001 and -7.6 [95% CI: -10.9 to –4.3] ml/100g/min per mmol/l, r = -0.11, p<0.001, respectively). The influence of hemoglobin on CBF in voxel based analysis of ASL data is shown in Fig 2.

## Discussion

In the present study we have examined the association of cognitive function change with regional and global CBF in middle-aged asymptomatic men with and without subclinical cognitive decline adjusting for other vascular risk factors.

The main findings are that sub-clinical loss of cognitive function is associated with hypoperfusion in the posterior cingulate and precuneal areas, but not with global perfusion. In addition, the analysis showed that this association appeared to be independent from regional brain atrophy and of vascular risk factors, although the association of cognition with regional CBF could not be statistically separated from that of global atrophy.

The present study is unique by including non-demented, asymptomatic participants with apparent sub-clinical cognitive decline. Most previous studies investigating the relationship between regional CBF and milder degrees of cognitive impairment have mainly focused on patients with subjective cognitive difficulties in terms of early Alzheimer’s disease or mild cognitive impairment. In such persons, the classical Alzheimer’s disease pattern of reduced perfusion (or metabolism) in the posterior cingulate, precuneus, hippocampal and parieto-temporal cortices are often reported [30]. The present finding of CBF reductions in the cingulate and precuneus appears to be in line with these observations and suggests that these characteristic CBF changes may develop even before any subjective cognitive difficulties arise. Similarly, characteristic regional CBF changes have been reported in asymptomatic persons with a family history or genetic disposition to dementia [5–7]. Notably, the two groups did not differ with respect to APOE4 genotype which has been associated with CBF changes in the hippocampal regions [6;31]. Whether similar hippocampal hypoperfusion was also present in this study cohort cannot be determined due to the lack of coverage of the inferior parts of the brain. Full brain coverage would have been preferable, in particular when investigating the association of regional CBF changes with cognitive function. The choice of coverage was based on both the limited coverage of the QUASAR sequence and on the wish of minimizing the possible influence of flow velocities on absolute CBF quantitation [24].

An important issue when studying the aging brain is how to include age related atrophy in the analysis. Partial volume effects may influence results of low-resolution CBF measurements regardless of the method used, making it difficult to distinguish CBF decrease per imaging voxel due to loss of gray matter volume within the voxel from a “true” perfusion decrease in gray matter within the voxel. Several methods for partial volume error corrections have been proposed, but there is no general consensus on how such corrections should be performed. We have therefore relied on a more simple ROI based approach correcting for gray matter volume within the ROI. In that context it is noteworthy that the group effect was attenuated in voxel based analysis adjusting for BPF, but not in ROI based analysis adjusting for GM volume. This may be associated with the higher sensitivity of the ROI based analysis, but also shows that tissue atrophy may be partly responsible for the decrease in perfusion.

As shown in Fig 4, increasing BPF was associated with widespread CBF increase in a distribution very similar to the typical age related pattern of widened sulci, in particular in the parietal cortices also overlapping the precuneus and posterior cingulate regions. This could suggest that the group difference may represent a more advanced or degree of normal brain aging affecting regions of particular importance for cognition. This view is supported by a recent study showing that life-long physically active elderly individuals have relatively preserved CBF in the precuneus and posterior cingulate regions compared to sedentary age matched controls [32].

From a cross sectional study design it is difficult to conclude on the causalities of the associations of cognition, atrophy and perfusion; i.e. does cognitive decline lead to regional atrophy which in turn leads to a decrease in CBF or vice versa? These associations are complex and most likely bi-directional [15], as evidenced by a recent study reporting that age related regional reductions in CBF are independent from patterns of cortical atrophy [33].

Homocysteine was the only vascular risk factor associated with regional CBF changes in the precuneus and posterior cingulate. This observation is in line with previous studies showing an association of circulating homocysteine in healthy aged subjects [12] and of homocysteine lowering treatment in vitamin B12 deficient patients [34] with both global and regional CBF. Although the associations of homocysteine with cerebrovascular disease, brain aging and neurodegenerative disease are well established, the exact mechanisms are not clear.

Previous large population based studies have reported associations of various measures of global cerebral perfusion with vascular risk factors, but the findings are not entirely consistent with regard to individual risk factors [10;35]. Education (and other life style factors not investigated) could potentially also influence CBF and the findings presented here. We did not observe any influence on CBF, but the study sample may have been too small to detect such smaller risk estimates. It should also be stressed that the present analysis aimed at investigating the association of global and regional CBF with cognitive change, and that the possible effects of various covariates were controlled for in the initial model selection procedure as described. The present study also provides some insight into methodological aspects of the use of CBF measurements. Arterial spin labelling is widely used in studies of brain aging. As the ability of these techniques to quantitate CBF in absolute terms is debated, most studies rely on normalized, relative CBF maps. We used the a multi TI arterial spin labelling scheme [23] and applied model based analysis for absolute quantitation [16]. The present study confirms the ability of this approach to produce absolute CBF estimates in agreement with PCM measurements [24] which is further supported by the finding of an inverse association with hemoglobin. Analysis of relative CBF maps did not change the overall findings or interpretation of the study, but both the magnitude and extent of group differences were smaller using relative CBF maps. Absolute quantitation may thus increase the ability to detect more subtle effects, which may be particular important when taking into account also factors influencing global CBF, such as altered physiological states or aging. Indeed, the interpretation of aged related changes may depend on whether absolute of relative CBF measurements are analyzed [32].

Although CBF measurements using ASL identified relatively small regional CBF differences at the group level (approx. 10% difference between the two groups), the low signal-to–noise ratio of individual CBF maps limits the ability to detect such small regional CBF changes at the participant specific level. Pseudo-continuous ASL techniques may improve both signal-to-noise and coverage, and may in the future be used at the single individual in the clinical setting [36].

It appears that global and regional CBF measurements provide different information. Cognitive decline was associated with regional CBF reductions only, whereas global CBF was associated with global atrophy as measured by BPF. Although global CBF has previously been shown to be associated with structural brain aging in terms of atrophy, ischemic lesions and cognitive decline [9–11], longitudinal studies have failed to show an association of reduced total brain flow with future brain volume loss, ischemic brain lesions and cognitive function change [15;37;38] suggesting that reduced global CBF is a consequence rather than a cause of brain aging. Still, global CBF may be considered a marker of cerebral health and function, and one recent study reported that global CBF is a powerful predictor of future overall mortality rate [39].

Documenting cognitive decline in a population with close to normal cognitive function may be difficult. The selection of participants for the two groups was based on the residual of each individual in a regressions analysis of the current and prior cognitive test scores of the entire population. As we expected only rather small changes in cognitive function, we chose to include only the extreme ends of the spectrum in order to obtain a larger exposure range with a relatively small sample. In the present analysis cognitive change was dichotomized for simplicity, and sensitivity analyses showed that analyzing continuous outcomes (residuals or late life cognitive score adjusted for early life cognitive score) did not change the overall results or interpretation.

Ideally, the participants would have been subjected to the same test at follow up, but the BPP test used at military draft exam is classified and was not available. The IST test was chosen for its similarity with BPP and the test scores were also highly correlated. Group A and B did not differ at baseline BPP scores, and the additional cognitive testing confirmed highly significant differences in current cognitive function of the two groups, suggesting that the group differences reflect changes in cognitive function rather than random measurement error. We therefore believe that although cognitive changes may be difficult to assess at the individual level, the group differences at follow-up can most likely be attributed to a true cognitive decline at the group level.

It should also be pointed out that none of the participants had any subjective cognitive difficulties and accordingly did not fulfil the criteria for mild cognitive impairment. Demographically, the two groups differed at young age only with respect to education. In that perspective, it is intriguing if the longer education length in Group A reflects higher cognitive function (or educational skills) not revealed by the BPP test, or if longer education serves to prevent cognitive decline as suggested by other studies [40]. However, the mechanisms explaining effects of education are not clear. Higher educational attainments may act by providing mental tools and strategies for coping with early cognitive dysfunction rather than by protecting against neurodegenerative processes. Furthermore, the study was designed to focus on associations with cognitive change and not with cognitive function at the time of follow-up. Accordingly, the observed group differences in regional CBF were not attenuated by adjusting for education.

In conclusion, the present study shows that sub-clinical cognitive decline is associated with specific regional CBF patterns in regions known to be associated with cognitive dysfunction. Conversely, cognitive decline was not associated with global hypoperfusion. The effects on regional CBF appear to be unrelated to vascular risk factors and regional atrophy, but cannot be confidently separated from normal age related brain atrophy.
